# My 15-Year Journey with *Nano Letters*

**DOI:** 10.1021/acs.nanolett.5c04525

**Published:** 2025-10-20

**Authors:** Fei Ding

**Affiliations:** Institut für Festkörperphysik, 26555 Leibniz Universität Hannover, Appelstraße 2, 30167 Hannover, Germany

For me, *Nano Letters* is not merely a venue for publishing results, it’s more like an old friend and a platform that documents our efforts along the journey.

## How It Started: Strain Tuning of Nanostructures

My relationship with *Nano Letters* began during the final stages of my doctoral studies. I was working on an experiment involving strain control of semiconductor quantum dots (QDs) and also graphene. The goal was to apply biaxial tension to the two-dimensional nanomembranes and thin films using piezoelectric materials. We chose the so-called PMN–PT (lead magnesium niobate-lead titanate) to obtain a large strain tuning range, especially at low temperatures. Initially, this approach was far from mature, and we spent considerable time addressing technical challenges related to graphene fabrication, device adhesion, symmetric stretching, and signal detection. Experiments were often difficult to reproduce.

Eventually, we discovered that this method allowed us to study the effects of strain on QD’s and graphene’s band structures with remarkable precision. At that time (year 2009), graphene was an extremely hot material, but the community lacked mature experimental approaches for systematically controlling its physical properties by strain. The most common way was to put graphene on a bendable substrate, but it is not suitable for many experiment configurations. With our method (Figure 1a), it is possible to apply controllable strain to graphene with highly controlled steps.^1^ Moreover, this method is compatible with complex experiments (transport and optical investigations). Therefore, we decided to submit it to *Nano Letters*.

**Figure 1:**
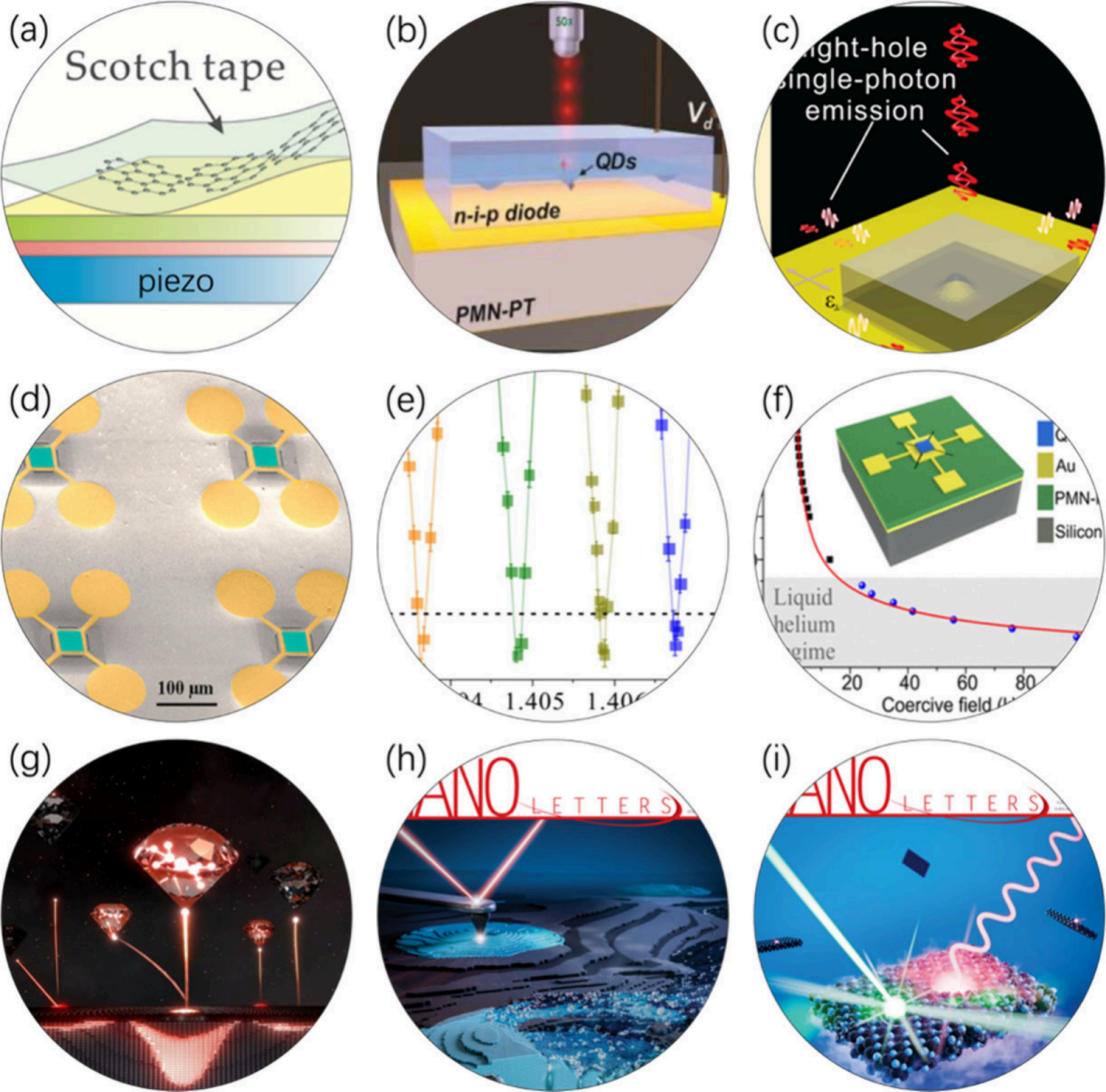
(a) Graphene layer was transferred onto a piezoelectric substrate, a simple device that enabled detailed studies on strained graphene. (b–f) Various experiments on the strain tuning of semiconductor quantum dots, leveraging the technical expertise accumulated from our graphene strain work. (g–i) Our recent works on the exploration of single quantum emitters, with (h) and (i) published as cover highlight. Reproduced from refs 1–9 . Copyright American Chemical Society.

I knew by that time *Nano Letters* was a prestigious journal. So, after submission, I felt quite anxious. I had no prior experience publishing in high impact journals. Fortunately, the reviewers provided constructive feedback and recognized our efforts in experimental design. The moment our paper was accepted, I experienced for the first time the profound joy of being truly ″heard″ as a young researcher.

What surprised me even more was that, thanks to the broad audience of *Nano Letters*, this paper and our proposed piezoelectric control method were subsequently cited by many colleagues and applied to various two-dimensional materials and device studies. Even today, researchers continue to extend this approach, generating many interesting results. For me, this *Nano Letters* publication was not only a milestone of my doctoral work but also the gateway through which I truly entered the research community.

## How It Continued: Solid-State Quantum Light sources

After the initial graphene strain work, my connection with *Nano Letters* never ceased. As my research evolved as a postdoc and then a junior group leader, I gradually shifted focus more toward semiconductor QDs, with the aim to transform these tiny ″artificial atoms″ into controllable quantum light sources. During this period, leveraging the technical expertise accumulated from our graphene strain work, we successfully implemented strain engineering on semiconductor QDs and produced several compelling studies (Figure 1b–f): tuning single-photon emission wavelengths using QD embedded in a diode structure,^2^ modulating light-hole exciton emission through strain engineering,^3^ and controlling entangled photon emission from self-assembled quantum dots via electric fields.^4^ These papers were subsequently published in *Nano Letters*, and we felt a profound sense of accomplishment.

Among these works, what impressed me most was combining microelectromechanical systems (MEMS) with semiconductor QDs to achieve chip-scale strain control.^5^ This idea initially seemed somewhat risky and proved very challenging to implement, but when we finally saw quantum dots being precisely stretched on-chip, I realized this represented a truly ″application-oriented″ direction. We later used quantum dots as strain sensors to measure temperature-dependent coercive field.^6^ For such interdisciplinary nanoscale research, *Nano Letters* was undoubtedly one of the ideal publication platforms.

In recent years, our collaboration with *Nano Letters* has continued (Figure 1g–i). Notable examples include “A Solid-State Source of Single and Entangled Photons at Diamond SiV-Center Transitions Operating at 80 K”,^7^ which demonstrated quantum light sources with emissions match that of the SiV-center in diamond; “Unveiling the 3D Morphology of Epitaxial GaAs/AlGaAs Quantum Dots”, which involved three-dimensional morphological measurements of quantum dots;^8^ and “Submillielectronvolt Line Widths in Polarized Low-Temperature Photoluminescence of 2D PbS Nanoplatelets”, investigating the photoluminescence properties of two-dimensional lead sulfide nanosheets.^9^ The latest two works (Figure 1h and i) were even highlighted on the covers. These topics all follow a consistent thread: how to transform nanostructures into stable and controllable quantum light sources. I’m delighted that these achievements could be shared on a platform like *Nano Letters*, and I’m particularly proud to see my students serving as first authors and even corresponding authors, documenting their efforts in this prestigious journal. These will be their starting points with *Nano Letters*.

## Looking Ahead

Looking back, my 15-year story with *Nano Letters* may represent of a typical example of a young scholar’s research journey: from nervously submitting the first paper to a high impact journal, to gradually exploring and establishing new directions, to now accompanying students through submissions, reviewer comments, and iterative revisions. Each acceptance in *Nano Letters* brought excitement, and each rejection taught us to pay great attention to detail.

Looking ahead, our future research will undoubtedly bring new (nano)­materials, novel physical mechanisms, and fresh challenges. For me, *Nano Letters* is not merely a venue for publishing results, it’s more like an old friend and a platform that documents our efforts along the journey. It has witnessed the moment when young researchers are first “seen” by the broad audience and observed how a field gradually accumulates from small exploration ideas (in our example, the strain tuning of QDs and nanomaterials) into an established methodology in the fields.

I believe *Nano Letters* will continue to maintain its high impact and forward-thinking perspective. It will continue to serve as a stage for both young and established researchers. My group hopes to continue submitting our best works here, and I hope more young people can find their own starting point here, just as I did 15 years ago.
